# Impact of weak radiofrequency and static magnetic fields on key signaling molecules, intracellular pH, membrane potential, and cell growth in HT-1080 fibrosarcoma cells

**DOI:** 10.1038/s41598-023-41167-5

**Published:** 2023-08-30

**Authors:** Hakki Gurhan, Frank Barnes

**Affiliations:** Department of Electrical, Computer and Energy Engineering, University of Colorado at Boulder, 425 UCB #1B55, Boulder, CO 80309 USA

**Keywords:** Cancer, Biomedical engineering, Quantum physics

## Abstract

There are substantial concerns that extended exposures to weak radiofrequency (RF) fields can lead to adverse health effects. In this study, HT-1080 fibrosarcoma cells were simultaneously exposed to a static magnetic flux density between 10 $\mathrm{\mu T}$ and 300 $\mathrm{\mu T}$ and RF magnetic fields with amplitudes ranging from 1 nT to 1.5 μT in the frequency range from 1.8 to 7.2 MHz for four days. Cell growth rates, intracellular pH, hydrogen peroxide, peroxynitrite, membrane potential and mitochondrial calcium were measured. Results were dependent on carrier frequency and the magnitude of the RF magnetic field, modulation frequencies and the background static magnetic field (SMF). Iron sulphur (Fe-S) clusters are essential for the generation of reactive oxygen species and reactive nitrogen species (ROS and RNS). We believe the observed changes are associated with hyperfine couplings between the chemically active electrons and nuclear spins. Controlling external magnetic fields may have important clinical implications on aging, cancer, arthritis, and Alzheimer’s.

## Introduction

Weak magnetic fields have been demonstrated to modulate cancer cell growth rates, alter the concentrations of reactive oxygen species (ROS) and reactive nitrogen species (RNS)^1–4^. The objective of this paper is to explore specific chemical reactions that can potentially be influenced by magnetic fields, thereby enhancing our understanding of how these alterations interact with biological systems and lead to additional changes in biological responses. It is anticipated the initial modifications will involve the coupling of the magnetic field with molecules or ions possessing magnetic dipole moments. Such entities may encompass radicals or molecules featuring unpaired electrons, nuclear magnetic moments, or electron transport proteins that contain transition metal ions such as iron and copper. By investigating these interactions, we aim to shed light on the underlying mechanisms and provide insights into the broader implications for magnetic field-mediated effects on biological systems.

### Radiofrequency magnetic fields interactions with biological systems

A growing body of research has provided evidence that weak radiofrequency (RF) magnetic fields can influence the concentration of ROS in living organisms^5,6^. Consequently, it is now widely acknowledged that biological systems can detect and be modified by weak magnetic fields. The influence of RF magnetic fields on biological systems has been firmly established^7,8^. In line with the increasing number of studies in this field, the present work encompasses experimental findings concerning the effects of RF fields on HT-1080 fibrosarcoma cells. Moreover, this paper proposes a mechanism through which an RF magnetic field with nano-Tesla (nT) intensity can alter growth rates and the production rate of ROS and RNS, drawing upon the previously discovered phenomenon known as the "Radical Pair Mechanism"^9^. These findings significantly contribute to the understanding of the intricate interplay between RF fields and biological systems, elucidating the underlying mechanisms involved.

In a low-intensity RF field, the energy of the interaction with electron magnetic moments is many orders of magnitude less than thermal fluctuation. Since the early observations^10,11^ concerning effects of the alternating weak magnetic field with frequencies between 1 and 10 MHz on living organisms, there have been ongoing efforts to explain how interaction with energy many orders of magnitudes below thermal energy are not being suppressed by thermal noise. It has been indicated the effect was caused by the interaction of RF fields with transition radical pairs, influencing the ROS formation rates through the radical pair mechanism^12^.

Radical recombination rates can be influenced by an applied static magnetic field (SMF) via the electron Zeeman interaction^13^. An RF field in combination with static magnetic fields (SMFs) can change the product yields if it is in resonance with the energy-level splitting arising from the hyperfine and Zeeman interactions^14,15^. Mitochondrial network may function as a frequency- and amplitude- modulated signaling system and may be sensitive to physiological variables such as rates of generation and scavenging of ROS.

**Figure 1:**
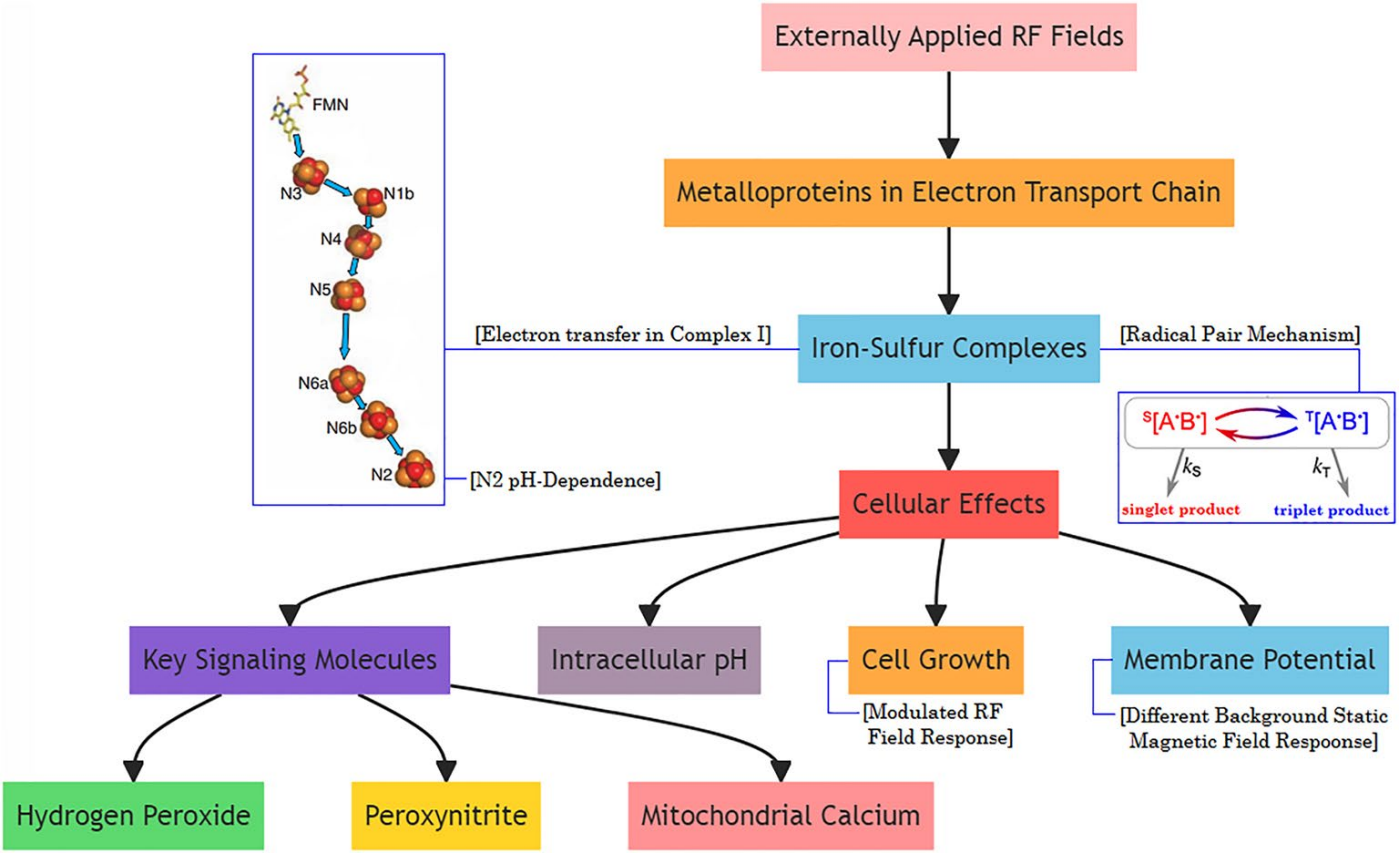
The interplay between externally applied RF fields, metalloproteins in the ETC, cellular effects, and key signaling molecules.

Complexes I and III of the electron transport chain (ETC) in mitochondria are thought to be the major sites of ROS production. Complex I is the largest and most complicated enzyme complex in the respiratory chain, and it also accommodates the greatest number of iron sulphur (Fe-S) clusters. Fe-S clusters are one of the major complexes and most Fe-S clusters are paramagnetic and have higher spin states. These proteins play roles in crucial processes such as cellular respiration. Although the Fe-S clusters are well known for their function as facilitating electron transport, they are also known to be involved in the active sites of many enzymes, performing functions such as initiation and stabilization of radical chain reactions. Different Fe-S clusters show hyperfine interactions with ^14^N of the protein between the frequencies 2.15 MHz and 3.85 MHz^16^. In this paper, we present a methodology for possible quantum effects by applied static and RF fields which induce changes in biological systems. Figure 1 illustrates the flow of information and highlights the different components involved in the study.

The redox potential (ΔE°) of a metal center can be explained as the tendency of the center to gain electrons to get reduced or lose electrons to get oxidized. The more positive the value of ΔE°, referred to as high ΔE° centers, the more favorable the reduction of the metal center becomes, and thus they are called oxidants. In addition, centers with less positive values of $\mathrm{\Delta E}^\circ$ are called low ΔE° centers and their oxidation are more favorable, so they are called reductants^17^. The rate of electron transport is affected by several factors, based on the Marcus equation:

1 $$ {\text{k}}_{{{\text{ET}}}} = \frac{2\pi }{{\text{h}}}\left| {{\text{H}}_{{{\text{DA}}}} } \right|^{2} \frac{1}{{\sqrt {4\pi \lambda k_{B} T} }}\exp \left( { - \frac{{\left( {\lambda + \Delta G^{^\circ } } \right)^{2} }}{{4\lambda k_{B} T}}} \right) $$

where ${\text{k}}_{\text{ET}}$ is the electron transfer rate, ${\text{H}}_{\text{DA}}$ is the electron coupling between the electron donor and electron acceptor, $\lambda$ is the reorganization energy, and $\Delta$G° is the total Gibbs free energy, called the driving force^18^. Among the factors that influence the electron transport rate, $\mathrm{\Delta E}^\circ$ can play a significant role, as it provides the driving force ($\Delta$G°) for the ET reaction. Increasing the redox potential (ΔE°) would lead to a more negative value for ΔG°. This corresponds to a larger driving force for the electron transfer reaction, potentially resulting in an increased rate constant (${\text{k}}_{\text{ET}}$). Decreasing the redox potential (ΔE°) would lead to a less negative (or even positive) value for ΔG°. This corresponds to a reduced driving force for the electron transfer reaction, potentially resulting in a decreased rate constant (${\text{k}}_{\text{ET}}$). By tuning $\mathrm{\Delta E}^\circ$ of different metal centers, the ET rate can be accurately controlled^19^. Electron transfer reactions in mitochondrial respiration is a good example of such tuning.

Complex I is the largest and first enzyme complex in the ETC of mitochondria. Its primary function is to facilitate the transfer of electrons from NADH (nicotinamide adenine dinucleotide) to ubiquinone (coenzyme Q) over a distance of approximately 9 nm. This electron transfer process is facilitated by the utilization of flavin mononucleotide (FMN) and seven iron-sulfur (Fe-S) clusters, namely N3, N1b, N4, N5, N6a, N6b, and N2^20^. The transfer of electrons within Complex I is essential for ATP production.

For instance, the electron transfer rate between the iron-sulfur clusters N6b and N2 is estimated to be $1.8\times {10}^{6}$ s ^(−1)^^21^. In the ETC, as electrons pass through the carriers, protons are actively pumped across the membrane against their concentration gradient. This process establishes a proton gradient, which plays a vital role in ATP synthesis and other energy-dependent reactions.

The pH dependence of the midpoint redox potential of N2, in conjunction with its spin–spin interaction with ubiquinone radicals^22^, suggests that changes in pH levels induced by externally applied RF fields can effectively modulate the potential of N2. If the redox potential of N2 decreases, it implies that the electron transfer from N6b to N2 becomes more favorable. This can lead to an increase in the redox potential between N6b and N2. In the context of the system described, a decrease in the redox potential of N2 would result in a larger driving force for electron transfer from N6b to N2, facilitating the overall electron transport process. This highlights the significance of the electron transport from N6b to N2.

### Basic concepts of radical pair mechanism

A radical is defined as a molecule that having an unpaired electron and thus a magnetic moment. Radical pairs may be generated when a bond is cleaved homolytically or an electron transfer takes place between non-radical species so that both molecules have an odd number of electrons and a magnetic moment^13,23^. When the spins are aligned so that their magnetic moments are antiparallel, they are said to be in an S or singlet state. If their spins are parallel, then they are in a T or triplet state meaning that, in magnetic fields, there are three energy levels as shown in Fig. 1a. Similarly, an additional set of energy levels are formed when there are an odd number of nuclear spins so that each of these energy states may be further divided into four more levels. However, the magnetic moments generated by the nuclear spins are typically about 1800 times smaller than those generated by electron spins.

### Zeeman effect

In the presence of an external magnetic field, the two electrons may have components of both of their spins—either parallel to it (the T_+_ state) or antiparallel to it (the T_-_ state). These are both magnetic in the direction of the field but, in the perpendicular directions the spin components average to zero. There’s a third triplet state, T_0,_ in which the spins are parallel to each other, but perpendicular to the externally applied field. In the singlet or S state, the spins of the two electrons are antiparallel and the magnetic moments cancel out each other^13^.

**Figure 2:**
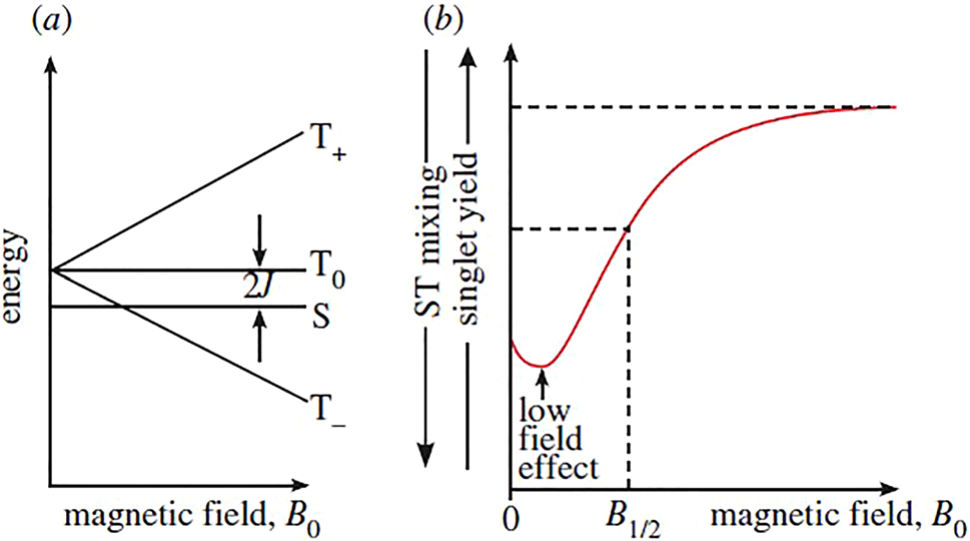
Energy level diagram (**a**) Magnetic field vs energy (**b**) Magnetic field vs singlet yield (fraction of excited state molecules that undergo a transition to the singlet state)^24^.

At zero field all three triplet states are of equal energy (are degenerate) but may be removed to the singlet state by the electron exchange interaction (2 J) which originates in the electrostatic interactions between the electrons. When an external magnetic field is applied, the energies of the two magnetic states, T_+_ and T_−_ states split from T_o_ state equally but with opposite relative signs. Therefore, (T_+_, T_−_) diverges with respect to T_o_ as the magnetic field, B_o_ increases. This is called the Zeeman Effect (Fig. 2). The energies of the other two states (S and T_o_) are unaffected by increase in B_o_.

The interaction of a spin with a magnetic field is named Zeeman interaction. The energy ∆E of the Zeeman interaction of an electron in a magnetic field of strength B is given by:

2 $$\Delta E=hv=g\mu B\times B$$

where h is Planck’s constant, ν is the frequency, $g$ is the Landé g-factor, $\mu B$ is the Bohr magneton, B is the magnetic flux density, and × denotes multiplication.

Fe-S clusters are known for their ability to undergo reversible redox reactions, allowing them to participate in electron transfer reactions. In a redox reaction, a species can be either oxidized or reduced. In many iron-sulfur clusters, the iron ions are predominantly found in either the Fe^2+^ or Fe^3+^ oxidation state. For example, The N2 cluster is an essential component of complex I and plays a crucial role in the transfer of electrons during the respiratory chain. It consists of four iron atoms and four inorganic sulfur atoms, forming a 4Fe-4S cubane-like structure. The N2 cluster commonly exists in the Fe^2+^ oxidation state. All four iron ions within the cluster are typically in the Fe^2+^ state, and they are coordinated with sulfur ions to form a stable cluster structure. To calculate the Zeeman shift for a N2 cluster with the given parameters, we can use the Zeeman equation.

Landé g-factor represents the proportionality between the magnetic moment and the spin angular momentum of a particle in the presence of a magnetic field. It characterizes the interaction of the spin with the magnetic field. It is a dimensionless constant specific to the particle under consideration. The numerical value of the g constant varies depending on the system and the nature of the particle. For the N2 cluster, the Fe^2+^ ions have a g-factor of 1.94^16^, and the Bohr magneton (μB) is approximately 4.9 μB^25^. When the magnetic field strength (B) is 45 micro-Tesla (µT), the energy shift (ΔE) for the N2 cluster, considering the presence of 4 iron ions, is approximately 7.875 × 10^–9^ eV. In addition, the energy change when the magnetic field changes from 10 µT to 300 µT for the N2 cluster is approximately 28.3896 × 10^–6^ eV. Even a small energy difference can affect the relative stability or population of different spin states, which can have implications for the cluster's reactivity, magnetism, or other properties. Understanding the magnitude of the Zeeman shift can contribute to characterizing and predicting the cluster's behavior under different conditions.

### Hyperfine interaction

There are magnetic interactions between the earth’s magnetic field and the spins of the nuclei of certain atoms. Atomic nuclei with an odd number of protons or neutrons have spin angular momentum and therefore magnetic moments. Those magnetic moments are typically about eighteen hundred times weaker than those of electrons, but because of the remarkably close distances, they generate magnetic fields that interact with the magnetic moments of the electron. Interactions between the magnetic moment of an unpaired electron and the nuclear magnetic moments (a few nearby bonded nucleus) are called hyperfine interactions. This interaction between the unpaired electron and the nuclei of certain atoms provides a mechanism to interconvert both electron and nuclear spin quantum numbers in the absence of an external magnetic field^26^.

### Radical pair reaction by electron transport

Electron transfer reactions are commonly affiliated with radical pair dynamics since radical pairs are repeatedly created and abolished through electron transfer^12^. Interconversion and chemical fates of the S and T states can be influenced by external fields. An externally applied magnetic field can change the yield by regulating competition between S and T states. If the interconversion between S and T is deprived by the external magnetic field, then less yield will be produced, and more radicals will recombine. If the external field enhances S − T interconversion, fewer radicals will recombine^27^. Singlet state allows for recombination which yields reduced ROS levels. On the other hand, triplet state allows for diffusion of radicals which yields increased ROS levels^28^. It has been shown that, in principle, applied weak magnetic fields can raise the yield of free radicals and abolish recombination between 10 and 40%^29^.

Radicals formed in electron transfer proteins can change the chemical fate of these proteins and in turn can modify the rate at which the electrons move to the next electron transfer protein^30^. This has a direct effect on parameters measured such as cell growth, intracellular pH, and membrane potential. Identifying the steps where changes in the magnetic field change the rate at which signaling molecules (such as hydrogen peroxide) are generated in the long chain of reactions controlling the grow rate for the fibrosarcoma cell are also a major long-term goal of our project.

### Electron flow through metalloproteins

Redox reactions play a crucial role in almost all biological processes, including mitochondrial respiration, which is an important energy process. Biology utilizes redox reactive metal ions in these processes. Principally, the redox activity employs metal ions particularly qualified as biological cofactors. Although most metal ions are redox active, biology utilizes a limited number of them for electron transfer processes^31^. Cytochromes, Fe-S clusters, and copper-containing proteins are outstanding members of redox centers in electron transfer processes.

Fe–S clusters, which consist of iron and sulfur, are metalloproteins prevalent in almost all organisms^32^. They play a crucial role in energy conversion within living cells and are commonly found in large, membrane-bound complexes^16^. While other molecular components such as porphyrins may also be involved, this paper does not delve into that discussion.

Fe-S clusters are unique in that not only are they are paramagnetic with externally applied magnetic fields, but they are also pH dependent. Basically, redox potentials of these clusters change periodically to facilitate accepting or donating electrons^33^. Redox potentials of [4Fe-4S] iron sulphur complex approximately change − 60 mV per pH^34^. This dependence informs the analysis of how redox potentials may change with respect to applied fields in different frequencies in the RF region. Hydrogen bonds also play a significant role in modulating the reduction potentials of iron sulfur clusters. It’s shown a deletion of a direct hydrogen bond to a sulfur ligand can lower the cluster redox potential by increasing the electron density on this ligand^35^.

Concentrations of molecules or ions in each energy state can be modified by exposure to externally applied oscillating fields around hyperfine resonances. This change in concentrations of signaling molecules such as ROS and RNS can occur in a number of molecules. Additionally, shifts in hyperfine couplings change the hydrogen bond strengths and therefore the redox potential of Fe-S clusters. These clusters undergo oxidation–reduction reactions and both their oxidized and reduced states are paramagnetic. Fe-S clusters make bindings with different substrates to increase or decrease their redox potentials, which help them to accept or donate electrons.

Using the pH dependency of reduction potentials of these clusters, experiments have been conducted to determine whether changes in frequencies of external oscillating fields around hyperfine resonances can impact intracellular pH values. Changes in intracellular pH can potentially affect basically all cellular processes, along with membrane potential and cell growth^36^. Therefore, cell growth rates and membrane potential were also measured.

Intracellular pH is also related to mitochondrial function. Hydrogen peroxide (H_2_O_2_) and peroxynitrite (ONOO^-^) are important signaling molecules released from mitochondria, inducing oxidative stress. For that reason, we measured concentrations of hydrogen peroxide and peroxynitrite. In addition, mitochondrial calcium concentrations were measured with respect to different frequencies of externally applied RF fields.

## Results

**Figure 3:**
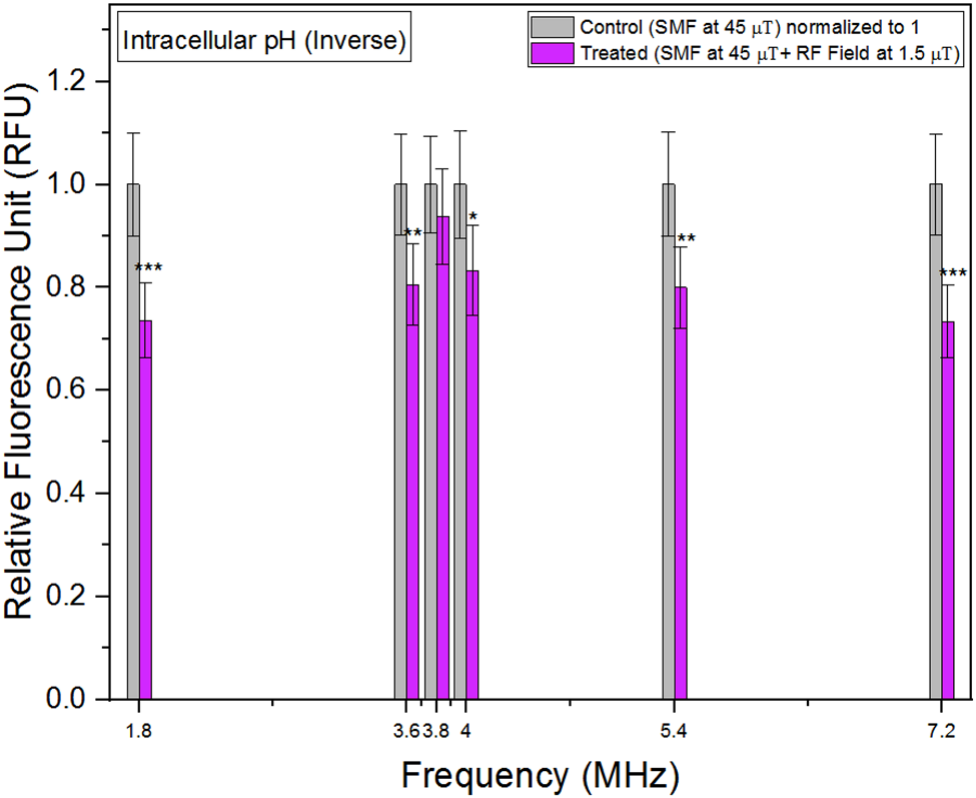
Intracellular pH (Inverse) concentrations as a function of frequency. The data are expressed as mean ± SD; n = 24 for each group.

**Figure 4:**
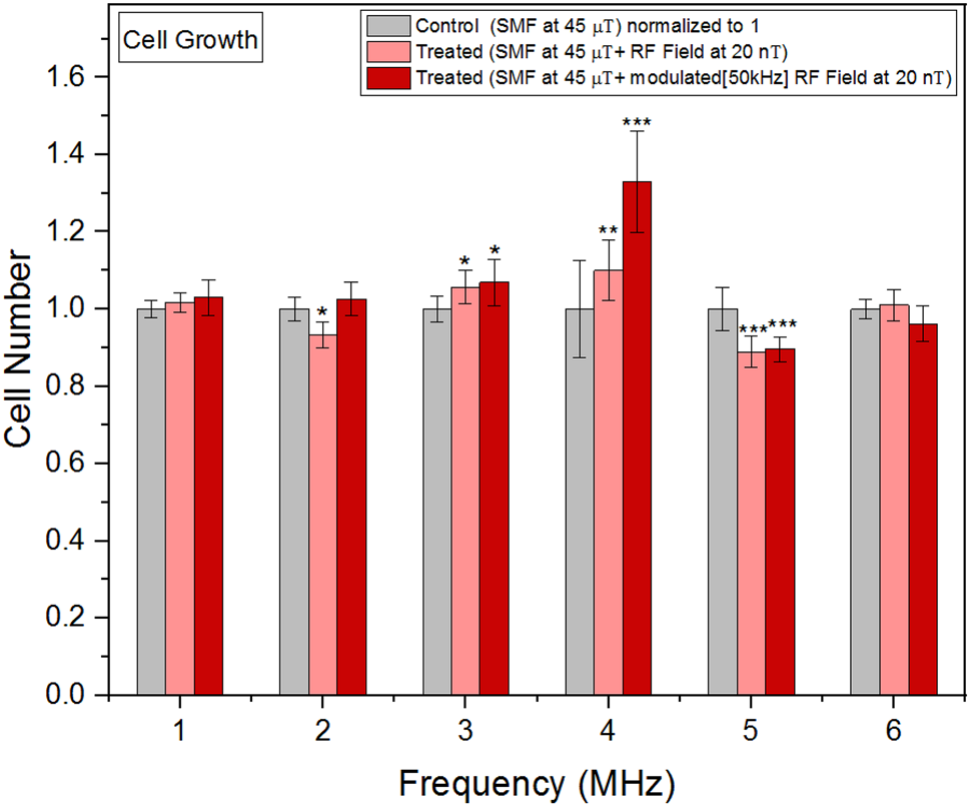
Cell growth rates as a function of frequency. The data are expressed as mean ± SD; n = 16 for each group.

**Figure 5:**
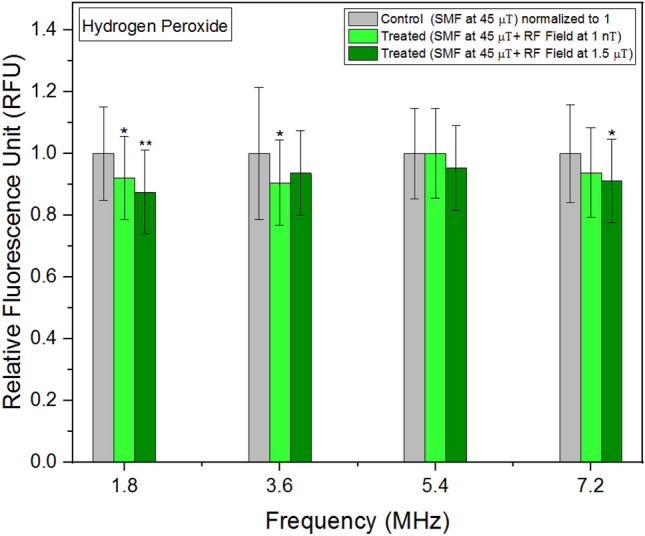
Hydrogen Peroxide concentrations as a function of frequency. The data are expressed as mean ± SD; n = 24 for each group.

**Figure 6:**
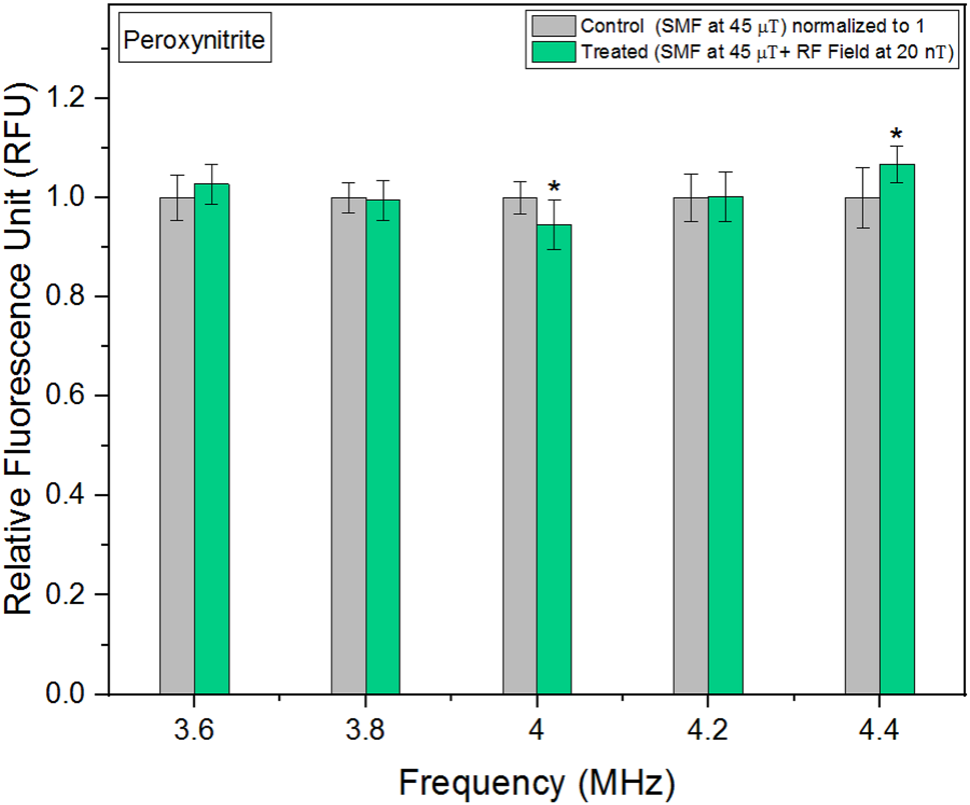
Peroxynitrite concentrations as a function of frequency. The data are expressed as mean ± SD; n = 24 for each group.

**Figure 7:**
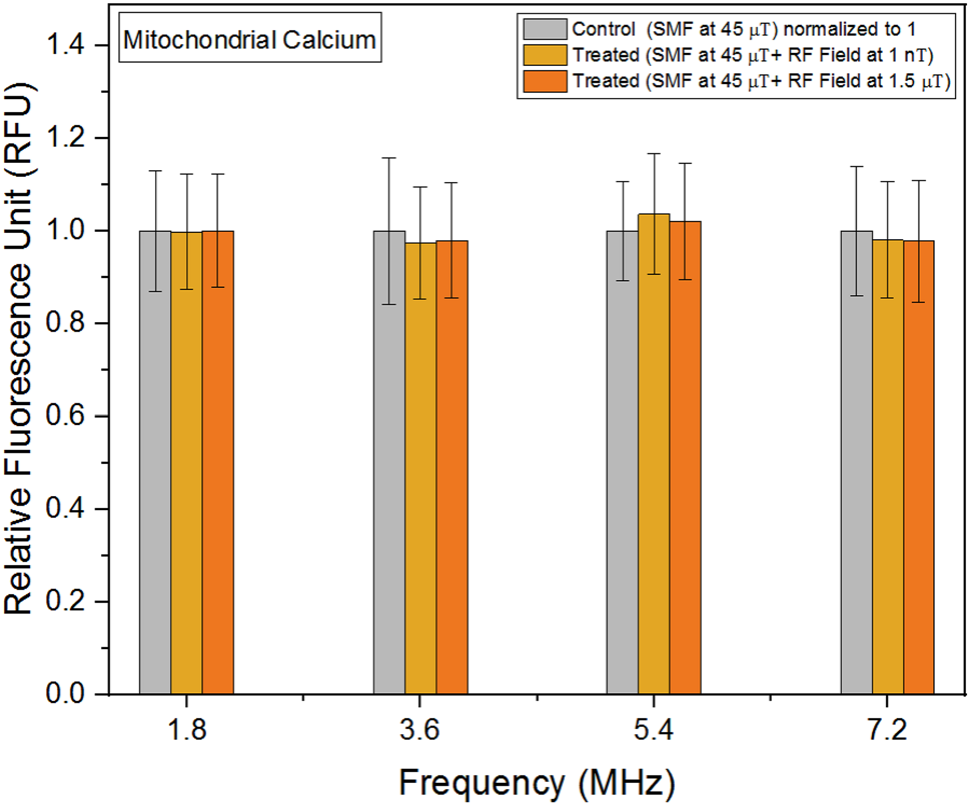
Mitochondrial calcium concentrations as a function of frequency. The data are expressed as mean ± SD; n = 24 for each group.

**Figure 8:**
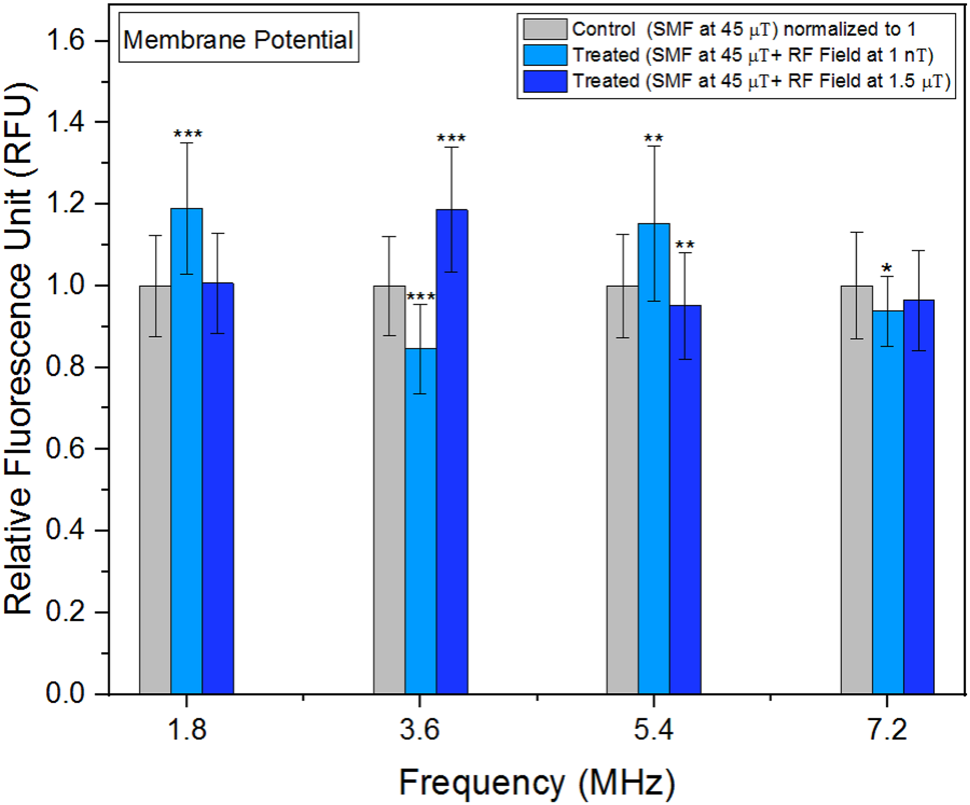
Membrane potential as a function of frequency. The data are expressed as mean ± SD; n = 24 for each group.

**Figure 9:**
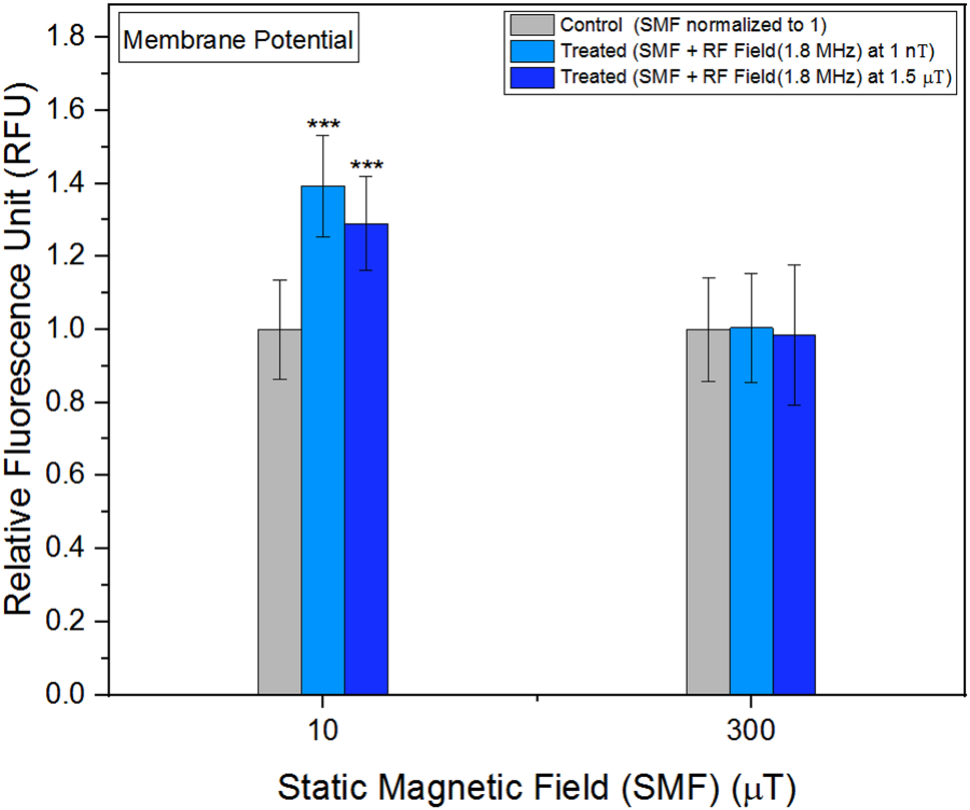
Membrane potential as a function background SMF. The data are expressed as mean ± SD; n = 24 for each group.

**Figure 10:**
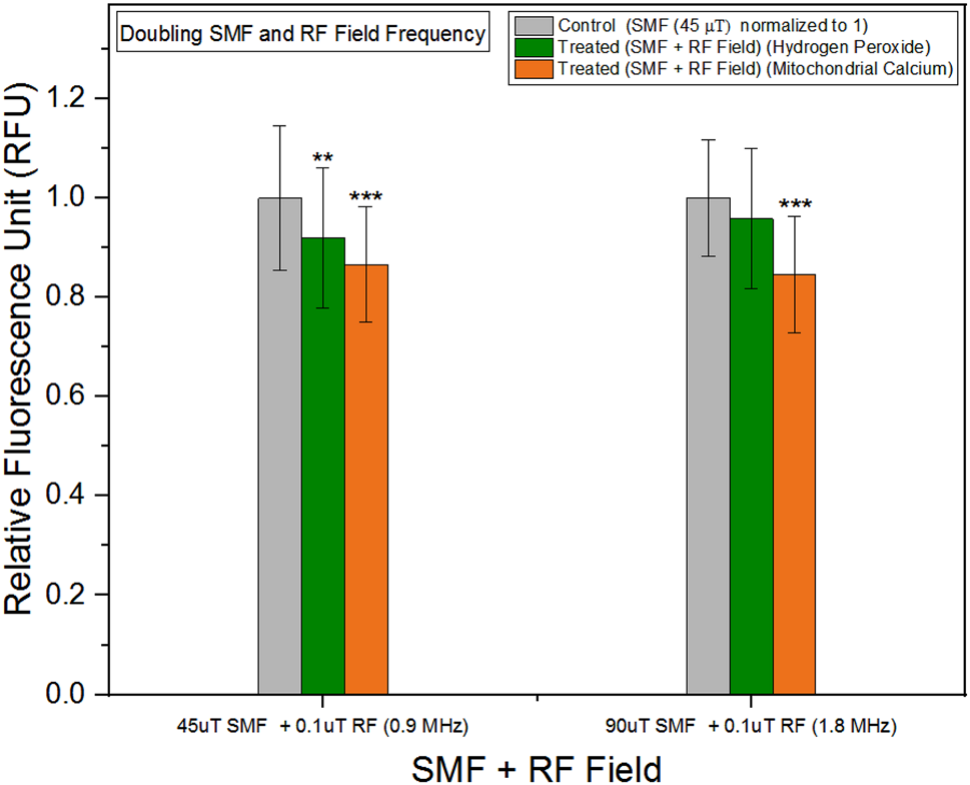
Hydrogen peroxide and mitochondrial calcium concentrations as a function background SMF and RF field. The data are expressed as mean ± SD; n = 24 for each group.

In Figs. 3, 4, 5, 6, 7, 8, 9, 10, we present the results of exposing HT-1080 fibrosarcoma cells to different intensities of SMFs and RF fields in parallel. Our study focused on investigating the effects of RF fields with frequencies ranging from 1.8 MHz to 7.2 MHz over a four-day exposure period. It is worth noting the molecular hyperfine coupling constant and the energy splitting of radical pairs and resonances in molecules are factors that influence the behavior of magnetic fields. These interactions typically occur in the frequency range of 0.1 to 10 MHz. Moreover, when radicals are formed by the reaction of singlet state molecules, they exhibit a low field feature at field strengths below 1 mT.

### Intracellular pH

In intracellular environments, the activity of hydrogen ions determines the intracellular pH value. When using fluorescent dyes such as pHrodo Green, the fluorescence intensity is influenced by the activity of hydrogen ions. These dyes are designed to emit stronger fluorescence at lower pH values, reflecting higher hydrogen ion activity. As a result, the relative fluorescent units (RFU) measured from these dyes are inversely related to the actual intracellular pH values. Higher RFU values indicate lower intracellular pH, while lower RFU values indicate higher intracellular pH.

Figure 3 shows the hydrogen ion activity (or relative fluorescent units representing hydrogen ion activity) in treated units is lower than in control units across all frequencies, indicating an overall increase in intracellular pH compared to the control units. Notably, the graph reveals the lowest increase in pH is observed between 3.6 MHz and 4 MHz, suggesting a potential association with the hyperfine resonances of Fe-S clusters.

### Cell growth

Figure 4 demonstrates the cell growth rates exhibited a nonlinear response when exposed to RF fields of the same intensities (20 nT) at various frequencies.

### Frequency modulation effects on cell growth

Our investigation focused on evaluating the impact of RF signals and modulated RF signals on cell growth rates across a range of frequencies, from 1 to 6 MHz. We specifically compared these effects with control conditions to gain insights into the potential biological responses to these electromagnetic stimuli.

In mitochondria, within Complex I, FMN acts as a cofactor. It accepts electrons from NADH and facilitates their transfer to the Fe-S clusters present in the enzyme. This electron transfer process relies on the redox potential difference between FMN and the iron-sulfur clusters. The iron-sulfur clusters serve as electron carriers, efficiently shuttling the electrons toward the final acceptor, ubiquinone. The redox potentials of the iron-sulfur clusters exhibit periodic changes, enabling the acceptance and donation of electrons during the enzymatic reactions. Considering the approximate FMN reduction time of 19 μs^37^, we selected a frequency modulation rate of 50 kHz, corresponding to a period of 20 μs. This choice allowed us to monitor the iron-sulfur cluster dynamics at a rate aligned well with the FMN reduction time, facilitating accurate analysis of their redox potential fluctuations.

Notably, our findings revealed both RF signals and modulated RF signals influenced cell growth rates. However, the analysis indicated that the modulated signals had a more pronounced effect on stimulating cell growth compared to RF signals alone. This observation suggests the 50 kHz modulation of the RF signals plays a crucial role in eliciting cellular responses.

Figure 4 below clearly illustrates the relationship between the frequency of the signals and the extent of change in cell growth rates. Notably, the most significant change in cell growth rates was observed at 4 MHz, indicating a frequency-specific response to the modulated RF signals.

### Hydrogen peroxide

When cells were exposed to RF fields at 1 nT and 1.5 μT across a wide range of frequencies, the mean values of hydrogen peroxide concentrations in the treated units were consistently lower compared to the control group (Fig. 5).

### Peroxynitrite

In addition to generating ROS, the mitochondrial respiratory chain is also capable of producing peroxynitrite, a potent oxidant^38^. RNS are oxidants derived from nitric oxide (NO). Initially, RNS were considered to be detrimental, but subsequent research revealed their role as efficient signal transducers^39^. In our study, when the background magnetic field was maintained at 45 μT and RF fields were applied in parallel at 20 nT, we observed a non-linear response. Notably, the lowest concentrations of peroxynitrite were observed at an RF field exposure of 4 MHz (Fig. 6). This finding is significant as moderate levels of peroxynitrite can serve as physiological signals for various cellular processes.

### Mitochondrial calcium

Mitochondrial calcium has been identified as a significant contributor to growth rates and various chemical parameters in experiments involving SMFs^1^. In our study, when the background magnetic field was maintained at 45 μT and RF fields were applied in parallel, we observed a minimal variation (less than 3%) in the concentrations of mitochondrial calcium across different frequencies (Fig. 7).

### Membrane potential

Experiments examining membrane potential yielded varied results when exposed to different frequencies and intensities of RF fields (Fig. 8). It is noteworthy the outcomes were particularly sensitive to the strength of the RF field, whether at 1 nT or 1.5 μT.

### Membrane potential in different background static magnetic fields

As previously mentioned in the context of the Zeeman Effect, it was anticipated various background SMFs would either amplify or reduce the energy gap between spin states. To investigate this phenomenon, RF fields were administered alongside different SMFs (Fig. 9). At a background SMF of 300 μT, the disparity between the membrane potential of the control and treated units diminished. Conversely, more substantial alterations (> 30%) were observed when the background SMF was set at 10 μT. Notably, a greater impact was observed at 1 nT, while a comparatively smaller effect was observed at 1.5 μT.

### Doubling SMF strength and RF field frequency

Equation (2) reveals a linear correlation between frequency and background magnetic field for the T+ and T− states. To investigate this relationship, we conducted experiments initially employing an SMF strength of 45 μT and an RF field frequency of 0.9 MHz. Subsequently, we measured the concentrations of hydrogen peroxide and mitochondrial calcium. In the subsequent phase, we doubled the SMF strength to 90 μT and increased the RF field frequency to 1.8 MHz, repeating the measurements of hydrogen peroxide and mitochondrial calcium concentrations. Figure 10, presented below, illustrates that, in both scenarios, the treated units exhibited similar responses.

## Discussion

In the frequency range of 1 MHz to 10 MHz, it has been proposed the mechanisms governing the magnetic effects on free radical reaction rates are influenced by quantum mechanical hyperfine resonances at the magnetic nuclei of specific molecules^40^. These resonances are sensitive to changes in the local magnetic environment, including the presence of external magnetic fields. By applying an external magnetic field, it is possible to alter the energy levels and transitions associated with the hyperfine structure of the nuclei, thereby modifying the resonance frequencies. The strength and orientation of the applied field can influence the splitting and alignment of the nuclear energy levels, affecting the resonant behavior. Thus, the variation in RF field amplitudes in these experiments allows for a comprehensive investigation of the relationship between the applied field strength, resonant behavior, and the specific phenomena being studied.

This research advances the argument that metalloproteins inside the ETC of mitochondria are some of the most important molecules to interact with externally applied static and RF magnetic fields. These electron transport proteins contain transition metal ions such as iron and copper. Iron and other ferromagnetic materials can create and maintain magnetic field. Unpaired electrons maintain magnetic moment approximately 600-fold greater than the magnetic moment of proton^41^. The more unpaired electrons in the atom or molecule mean the greater potential for these electrons to align their spin to externally applied magnetic fields. Presence of unpaired electrons within Fe-S clusters and their hyperfine resonances between 1 and 10 MHz make them possible molecules to interact with externally applied RF fields.

We investigated the effects of RF magnetic fields in combination with SMFs within the frequency range of 1.8 MHz to 7.2 MHz, considering the chemical properties of Fe-S clusters. Initial findings indicate intracellular pH levels exhibit changes corresponding to variations in frequencies within this range, suggesting a potential disruption of interconversion between the singlet and triplet states of the cluster near hyperfine resonances. Notably, cell growth rates exhibited a nonlinear response to frequency exposures between 1 and 6 MHz. Additionally, we employed a 50 kHz modulation of RF field amplitudes during exposure, resulting in increased cell growth rates across all applied RF frequencies. The most significant changes in cell growth rates were observed at 4 MHz.

Hydrogen peroxide and peroxynitrite, both vital signaling molecules, exhibited distinct behaviors in response to externally applied RF fields. The presence of RF fields resulted in a decrease in hydrogen peroxide levels, with the outcome slightly varying across different frequencies. Excessive hydrogen peroxide production has been implicated in diseases such as cancer and rheumatoid arthritis^42^, while low levels have been associated with cell proliferation^43^. Notably, Fe-S clusters play a crucial role in the generation of hydrogen peroxide^44^. In contrast, peroxynitrite demonstrated a nonlinear response to externally applied RF fields. Similar to intracellular pH experiments, the lowest concentrations were observed at 4 MHz. Different concentrations of peroxynitrite can either promote or inhibit various signaling pathways involved in cell metabolism, growth, and proliferation^45^.

No significant variations were observed in mitochondrial calcium concentrations following RF field exposures. While calcium uptake in mitochondria is governed by a membrane potential difference, it does not remain confined within the mitochondria^46^. In mammalian cells, mitochondrial stress is characterized by alterations in membrane potential^47^. Membrane potential represents the electrical potential difference between the interior and exterior of the plasma membrane. Our findings demonstrate both the amplitudes and frequencies of the RF fields influenced membrane potential values. Notably, these alterations in membrane potential were highly sensitive to the amplitudes of the relatively weak RF fields employed in our study. This underscores the significance of even subtle changes in RF field amplitudes in modulating membrane potential dynamics.

In addition to the aforementioned experiments, we conducted measurements of the membrane potential at different SMF exposures (10 μT and 300 μT), while keeping the RF field frequency fixed. The results indicated the increase in the background magnetic field at 300 μT did not yield a significant difference, potentially due to a larger energy gap between spin states, as governed by the Zeeman Effect. Furthermore, we examined the combined effects of the Zeeman Effect and hyperfine interactions by doubling both the frequency of the RF field and the amplitude of the SMF. In these experiments, we measured the concentrations of mitochondrial calcium and hydrogen peroxide. Remarkably, our findings align with the relationship between the background magnetic field and frequency described in Eq. (2), providing further support for the influence of these magnetic field parameters on the observed outcomes.

We proposed a mechanism by which externally applied RF fields can induce changes in mitochondrial chemistry by exploiting the hyperfine resonances of metalloproteins present in the ETC. The hyperfine resonant frequencies of these protein complexes allowed us to predict the appropriate RF ranges to be applied to HT-1080 human fibrosarcoma cells. Cellular mechanisms were reported to explain observed RF effects. Our results demonstrate RF treatment of cancer cells leads to significant alterations in mitochondrial activity, with effects varying depending on the specific frequencies and amplitudes of the RF fields. These findings highlight the capacity of RF fields to modulate mitochondrial function and suggest their potential as therapeutic tools for manipulating cellular activities in the context of cancer treatment.

## Methods

### Cell culture

HT-1080 human fibrosarcoma cells were cultured in Eagle's Minimum Essential Medium and supplemented with 10% fetal bovine serum. The cells were plated in a sterile tissue culture flask of 75 cm^2^ at a density of approximately 4000 cells per cm^2^. Once the cells reached 70–80% confluency, they were either transferred to flasks of 25 cm^2^ for cell growth experiments or to 12-well cell culture plates for fluorescence experiments. To promote optimum cell growth and maintain a healthy cell culture, temperature, CO_2_, and humidity variations were also controlled. The temperature of the incubator was set to 37 °C to mimic the human body temperature. The CO_2_ concentration in the incubator atmosphere was set to 5% to maintain the pH level of the cell culture media. A relative humidity of around 95% was achieved by placing 5 L of deionized water in a water pan inside the incubator. To prevent contamination of the cell culture, 50 mL of Aquaguard-1 (PromoCell GmbH) effective agent solution was added to the water tray per 5 L of water.

### Magnetic field stimulation and exposure setup

The experiments took place inside an incubator containing both a mu-metal shielding box and a configuration of Helmholtz coils. The purpose of incorporating a mu-metal shielding box within the incubator was to diminish any surrounding magnetic interference. This approach enabled precise control over SMFs and RF fields, thereby facilitating the achievement of consistent and replicable experimental conditions.

**Figure 11:**
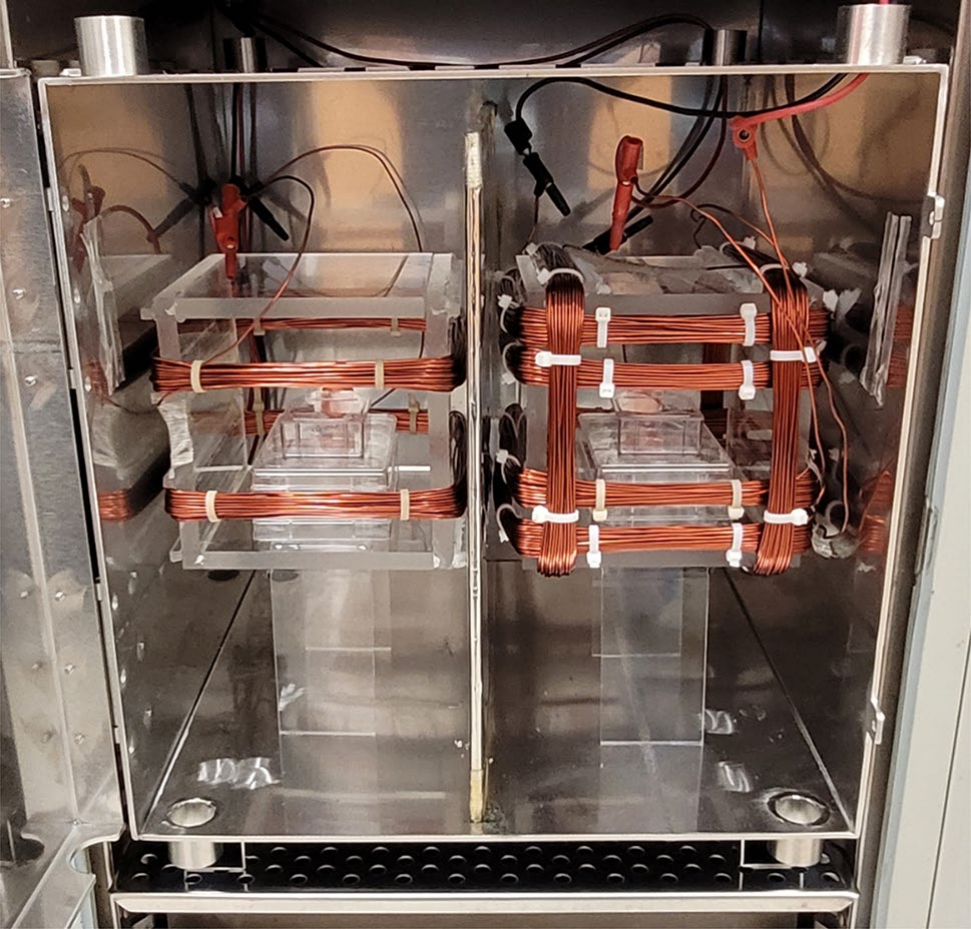
Exposure Setup with Mu Metal Separation and Coil Configuration. On the left side, a square Helmholtz coil is used for the control group, while on the right side, a square Helmholtz coil is used for the treated group. The inner coils of both the control and treated coils are responsible for generating an SMF, while the outer coils generate an RF field. The magnetic fields generated by the coils are oriented perpendicular to the growth surface. A mu metal sheet is placed between the control and treated coils, ensuring separation, and minimizing magnetic field interference between the two groups.

One set of Helmholtz coils was activated for the exposed cultures, while the other set, serving as the control, was separated by a mu metal sheet inside a mu metal cage (Fig. 11). The control cells in Helmholtz coils were maintained with a magnetic field of 45 µT and treated cells were exposed to different flux densities of magnetic fields. 14 AWG Copper Magnet Wire (Temco Industrial, MW0515) was used for the windings of the Helmholtz coils. This wire has several advantages in terms of physical moisture, chemical, and thermal resistance which contribute to the longevity, reliability, and safety of the Helmholtz coil.

The SMF measurements were conducted utilizing the FGM-4D2N fluxgate magnetometer from Walker Scientific, with a resolution of 0.1 µT. The homogeneity of the exposure fields exhibited variations of ± 5% within the area where culture flasks and well plates were positioned.

Some of the experiments were conducted by using the ferrite plate exposure system consisting of of four ferrite rods and two ferrite plates. The ferrite plates used in our study were sourced from Ferroxcube. These plates belong to the PLT core type and are made of 3C95 material. With an initial permeability (µi) of 3000, the plates have an uncoated finish. They measure 5.08 mm in thickness, 128 mm in length, and 101.60 mm in width. The ferrite rods used in our study were sourced from Fair-Rite Products Corp. These rods belong to the rod core type and are made of 78 material. With an initial permeability (µ_i_) of 2300, the rods have an uncoated finish. They have a diameter of 9.5 mm and a length of 70 mm.

**Figure 12:**
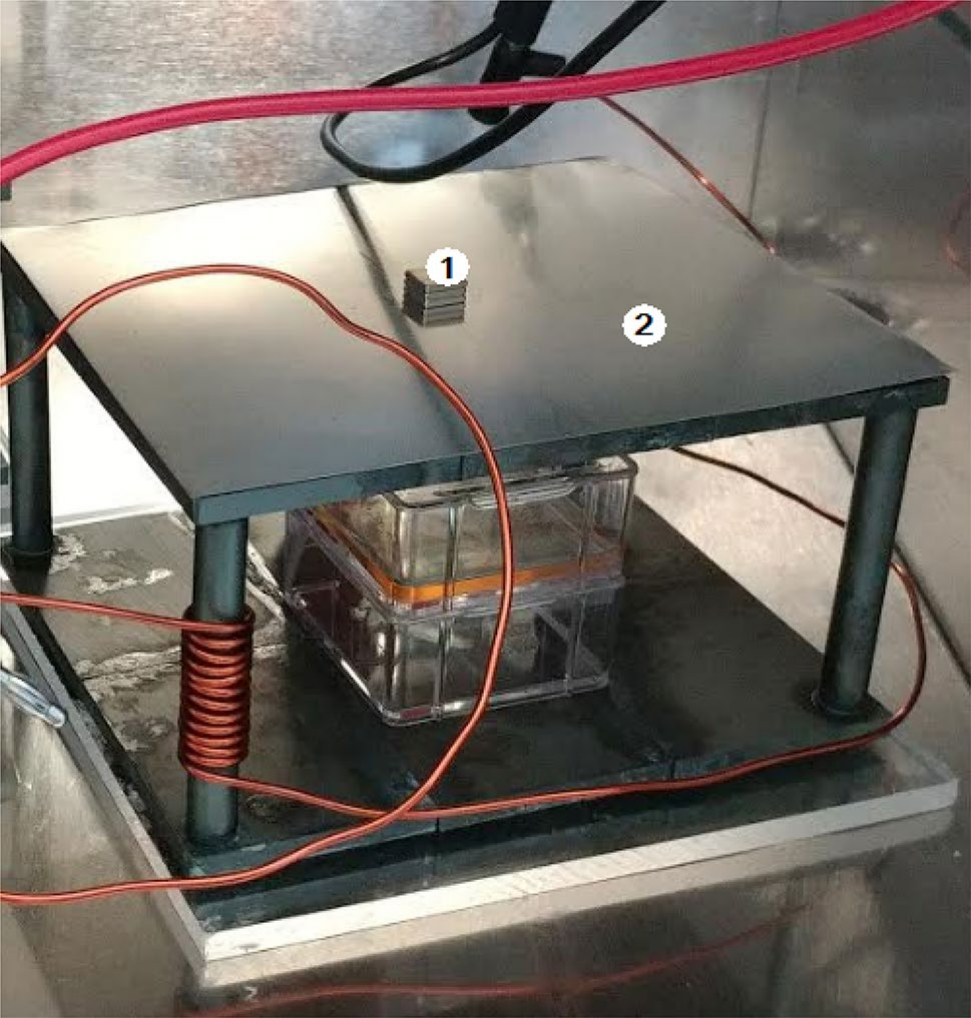
Ferrite Plate Exposure Setup with Permanent Magnets (1) and Mu Metal Sheet (2).

In our experimental setup, we connected the top and bottom ferrite plates using four ferrite rods positioned in the corners. SMFs generated by permanent magnets and a mu metal sheet was used to distribute these fields. To generate RF fields, 12 turns of wire were wound around two of the ferrite rods (Coil 1 and Coil 2) which have an effective permeability $({\mu }_{eff})$ of 29 and length of the coils around the rods was 45 mm. This ferrite plate exposure setup was employed in RF experiments where different background SMFs were applied. The Fig. 12 illustrates the experimental setup for ferrite plate exposure system.

Electromagnetic fields in the range between 1.8 MHz and 7.2 MHz were produced by an ICOM IC-7300 HF/50 MHz base transceiver and a function generator (Hewlett Packard, 33120A, 15 MHz arbitrary waveform generator). The transceiver was used to provide the maximum output (40 V peak-to-peak (pp)) for the strongest field condition and the function generator was used to provide the minimum output (50 mV pp) for the weakest field condition. We used frequencies available from the transceiver, specifically 1.8, 3.6, 5.4, and 7.2 MHz, to obtain enough periodic data points around expected hyperfine resonances of iron sulfur clusters. Additionally, we used a function generator to observe how these results changed with minimum input power. After discovering cell response to much lower magnetic fields (nano-Tesla), we conducted a more detailed scan of the 3–5 MHz region with additional data points generated by the function generator.

The magnetic field component between 1.8 MHz and 7.2 MHz and the electric field component within the same frequency range were measured using a loop probe (Beehive Electronics, Model 100C) and a stub probe (Beehive Electronics, Model 100D) respectively, both with calibrated sensitivity up to 3 GHz. In our study, we investigated the relationship between different magnetic field intensities and the corresponding induced electric fields. For the applied magnetic field intensities of 1 nT, 20 nT, and 1.5 µT, we subsequently measured the resulting electric fields in the medium. Our measurements revealed electric field values of 0.056 V/m, 1.128 V/m, and 84.68 V/m for the respective magnetic field intensities. Considering the medium's electrical conductivity of approximately 1.7 S/m, we calculated the corresponding Joule heating powers. The calculated values were approximately 0.0056 W/m^3^, 2.278 W/m^3^, and 2484.76 W/m^3^ for the electric fields of 0.056 V/m, 1.128 V/m, and 84.68 V/m, respectively.

Notably, the induced electric field of 84.68 V/m associated with a magnetic field intensity of 1.5 µT slightly exceeds the occupational and public exposure limits recommended by the International Commission on Non-Ionizing Radiation Protection (ICNIRP). These limits serve as guidelines to ensure safety in occupational and public settings. By providing these findings, our study contributes valuable insights into the relationship between magnetic fields and induced electric fields. Moreover, the identification of an induced electric field slightly exceeding the ICNIRP limits underscores the importance of considering safety measures and appropriate guidelines when evaluating the potential effects of electromagnetic fields on human health.

In addition, we employed J-type thermocouple wires to accurately measure the temperature, utilizing the DAQami software for efficient data acquisition and analysis. When measuring the temperature in our experimental setup, we found that the recorded values exhibited minimal fluctuations with a maximum change of only 0.4 °C. Notably, this measurement was obtained under the highest magnetic flux density condition of 1.5 µT. The limited temperature change suggests the influence of the magnetic field on thermal effects was relatively modest within our experimental setup. The limited variation in temperature suggests the randomizing effects of thermal energy were effectively controlled or minimized within our experimental system.

### Cell growth studies

In cell growth experiments, HT-1080 fibrosarcoma cells were cultured in Corning cell culture flasks (Corning Inc.) with a surface area of 25 cm^2^.To prevent genomic abnormalities and achieve enhanced proliferative capacity, cell culture passages ranging from three to 12 were utilized in the experiments. For the cell counting assay, two flasks (control and treated) were seeded with a concentration of 125,000 cells per flask and incubated at 37 °C in a 5% CO_2_ environment.

The effect of magnetic treatment on cell growth was determined by counting cell numbers after four days of stimulation. In this, we used two flasks, one for the samples treated with RF and SMF, and one for control (SMF only). Two flasks were taken from the incubator at the end of the fourth day. The culture medium was aspirated and the cells were rinsed with 5 mL of phosphate-buffered saline (PBS) per flask. Following the removal of PBS, 1.5 mL of trypsin was added to each flask. The flasks were placed in the incubator for 5 min, allowing the cells to detach from the bottom. Subsequently, the culture flasks were examined under a microscope to confirm detachment. To neutralize the trypsin, 4.5 mL of medium per flask was added. The cell solution was transferred to centrifuge tubes and centrifuged at 2000 rpm for 5 min using a XC-2000 Premiere centrifuge (C & A Scientific). The supernatant was carefully discarded, and 1 mL of culture medium was added to the pellet. Gentle pipetting was performed to achieve a homogeneous cell suspension. The cells were then diluted 1:1 in trypan blue. A mixture of 40 µL of the cell suspension and 40 µL of trypan blue was prepared and gently mixed. For each individual count, 10 µL of the prepared mixture was used.

Countess II Automated Cell Counter (Thermo Fisher Scientific) used this dilution to calculate the total cell concentration. The cell concentration, displayed on the screen, was directly expressed as cells per milliliter (cells/mL). A total of 16 samples were collected, and for each experimental condition, the mean and standard deviation were calculated based on the 16 measurements and presented.

### Fluorescence studies

Fluorescent assay measurements were conducted using a Corning 12-well plate, with a surface area of 3.85 cm^2^ per well. In the experiments, a total of 18 wells across two plates were seeded at a concentration of 5000 cells per cm^2^ and incubated at 37 °C in a 5% CO_2_ environment. Each well received 1 mL of the cell suspension. Among the wells, six were treated with different frequencies of RF fields in conjunction with a SMF of 45 µT, while the remaining six wells served as control and were exposed only to the SMF. The sample preparation protocol employed was the same as that used in the cell growth studies. Fluorescence studies were performed on the fourth day of continuous exposure to the static and RF magnetic fields. A multi-detection microplate reader, Synergy HT (BioTek Instruments), was utilized for the fluorescence measurements. The effects induced by the static and RF magnetic fields were examined in each experiment. A total of 24 measurements were taken per parameter, resulting in the presentation of mean and standard deviation values corresponding to the 24 measurements for each experimental condition.

### Intracellular pH

In the intracellular pH measurements, the pHrodo Green AM intracellular pH indicator from ThermoFisher Scientific was employed. To prepare the dye solution, 10 μL of pHrodo Green AM was combined with 100 μL of PowerLoad concentrate. This resulting mixture was further diluted with 10 mL of PBS. The growth medium was aspirated from the cells, which were subsequently rinsed once with PBS. The pHrodo Green AM/PowerLoad/PBS mixture was introduced to the cells, followed by an incubation period at 37 °C for 30 min to allow for dye uptake. Subsequently, the cells were subjected to analysis using a fluorescence plate reader with excitation at a maximum of 509 nm and emission at a maximum of 533 nm. This measurement enabled the assessment of intracellular pH levels.

### Membrane potential

The FluoVolt membrane potential kit obtained from ThermoFisher Scientific was employed for the experiment. To prepare a fresh FluoVolt loading solution, 12 μL of FluoVolt dye (1000X concentration), 120 μL of 100X PowerLoad concentrate, and 12 mL of PBS were combined in a 15 mL centrifuge tube and thoroughly mixed. For adherent cells, the growth medium was discarded, and the cells were washed twice with PBS. Subsequently, 1 mL of the FluoVolt loading solution was added to each well containing cells, and the cells were incubated at 37 °C for 30 min to allow for dye uptake. Prior to fluorescence measurements, the cells in the FluoVolt loading solution were washed twice with PBS to remove any unbound dye and suspended in PBS. The fluorescence intensity emitted by these cells was then quantified using a fluorescence plate reader, with appropriate wavelength settings of excitation at 522 nm and emission at 535 nm.

### Hydrogen peroxide

The Amplex Red reagent, obtained from ThermoFisher Scientific was utilized to quantify the production of hydrogen peroxide in HT-1080 fibrosarcoma cells. To create a stock solution of the Amplex Red reagent, the contents of the vial were dissolved in 60 μL of dimethyl sulfoxide (DMSO) to achieve a concentration of 10 mM. To prepare a 1X reaction buffer solution, 4 mL of 5X reaction buffer was combined with 16 mL of deionized water. The contents of the horseradish peroxidase (HRP) vial were dissolved in 1.0 mL of the 1X reaction buffer, producing a stock solution of HRP with a concentration of 10 U/mL. Next, 50 μL of the 10 mM Amplex Red reagent stock solution, 100 μL of the 10 U/mL HRP solution, and 4.85 mL of the 1X reaction buffer were mixed to create the Amplex Red reagent/HRP working solution. Subsequently, 500 μL of the working solution was added to each well of the microplate. Following the addition of the working solution, the samples were incubated at 37 °C for 30 min. Fluorescence measurements were taken using a fluorescence plate reader, with an excitation wavelength set at the maximum of 571 nm and an emission wavelength set at the maximum of 585 nm. These measurements allowed for the quantification of hydrogen peroxide levels in the samples.

### Peroxynitrite

In the peroxynitrite experiments, we utilized the Abcam Peroxynitrite Assay Kit (ab233468). To create a concentrated stock solution of Peroxynitrite Sensor Green, we added 20 μL of DMSO to the vial, resulting in a 500X concentration. To prepare the assay solution, we combined 10 μL of the 500X DMSO stock solution with 500 μL of assay buffer, ensuring thorough mixing. This resulting mixture was then added to the cells and incubated at 37 °C for a duration of one hour to allow for sufficient reaction. Following the incubation period, the cells were analyzed using a fluorescence plate reader, with excitation set at the maximum of 490 nm and emission set at the maximum of 530 nm. This measurement enabled us to accurately evaluate the levels of peroxynitrite within the cells.

### Mitochondrial calcium

In order to assess the mitochondrial Ca^2+^ levels in HT-1080 human fibrosarcoma cells, we utilized the Rhod-2 AM probe obtained from ThermoFisher Scientific. First, the cells were carefully washed and then suspended in phosphate-buffered saline (PBS) supplemented with Rhod-2 AM at a concentration of 1 μM. Following this, the cells were subjected to a 30-min incubation period at a temperature of 37 °C to allow for optimal probe loading. Before conducting the fluorescence measurements, we performed an additional wash step on the cells and suspended them once again in PBS. The resulting cells were then subjected to fluorescence analysis using a plate reader, where the appropriate wavelength settings were selected for excitation at 550 nm and emission at 580 nm. By following this protocol, we were able to accurately measure the fluorescence intensity emitted by the cells, providing valuable insights into the mitochondrial Ca^2+^ levels.

### Statistical analysis

All values were reported as the mean ± standard deviation. Statistical significance was assessed using the student's t-test followed by Tukey's post hoc test. P-values below 0.05 were considered statistically significant for differences observed in cell growth and fluorescence experiments. For normalization, each experimental group was compared to its corresponding control group, maintained at 45 µT, which represents the nominal Earth's magnetic field. In Figs. 3, 4, 5, 6, 7, 8, 9 and 10, asterisks (*) denote significance levels at P < 0.05, double asterisks (**) denote significance levels at P < 0.01, and triple asterisks (***) denote significance levels at P < 0.001. The statistical analysis of the data was performed using Origin Pro 2022 Statistical Package software (OriginLab Corporation).

## Data Availability

All data relevant to the study are included in the article. In addition, the datasets used and/or analyzed during the current study are available from the corresponding author on reasonable request.
